# 2068. Progress in the Infection Prevention and Control Monitoring System for the United States President’s Emergency Plan for AIDS Relief (PEPFAR) Programs and Supported Facilities

**DOI:** 10.1093/ofid/ofad500.138

**Published:** 2023-11-27

**Authors:** Danica J Gomes, Maria Insua, Mitesh Desai, Diana Forno Rodriguez, Deborah A Goldstein, Anand Date, Elizabeth A Bancroft, Catherine Godfrey, Nicole Zender

**Affiliations:** Centers for Disease Control and Prevention, Powder Springs, GA; Department of State, District of Columbia, District of Columbia; Department of State, District of Columbia, District of Columbia; U.S. Centers for Disease Control and Prevention, Atlanta, Georgia; USAID, Washington DC, District of Columbia; U.S. Centers for Disease Control and Prevention, Atlanta, Georgia; U.S. Centers for Disease Control and Prevention, Atlanta, Georgia; Department of State, District of Columbia, District of Columbia; Department of State, District of Columbia, District of Columbia

## Abstract

**Background:**

The U.S. President’s Emergency Plan for AIDS Relief (PEPFAR) prioritizes infection prevention and control (IPC) activities in its 40,000 supported facilities in over 50 countries. PEPFAR’s 2015 quality assurance tool included four IPC indicators (tuberculosis IPC [TBIPC], waste management [WM], injection safety [IS], laboratory biosafety [LB]). In 2022, four new IPC indicators were added (IPC program [IP], environmental cleaning [EC], availability of personal protective equipment [PPE], medical device reprocessing [MD]). As of October 2022, PEPFAR programs are required to assess IPC indicators in 10% of facilities over the course of one year. We report quarter one data results.

**Methods:**

Indicator data from October 1 - December 31, 2022 were analyzed. Scores were calculated based on compliance with 2-4 criteria each: green, yellow, and red represented good, fair, and poor compliance, respectively. Percentages of yellow and red scores were combined, to represent the percent of facilities requiring improvement.

**Results:**

A total of 272 sites were assessed across 15 countries; each PEPFAR program selects the indicators to assess in each site. For existing indicators (TBIPC, WM, IS, LB), 16%, 34%, 22%, and 57% of sites assessed scored red or yellow, respectively; for indicators added in 2022 (IP, EC, PPE, MD), 74%, 67%, 64%, and 61% scored red or yellow, respectively (Figure). Specific gaps include: 63% of facilities lack adequate IPC leadership; ∼50% lack adequate environmental cleaning staff, protocols, and methods; 41% lack respirators; 30% lack device reprocessing protocols.

**Figure:**
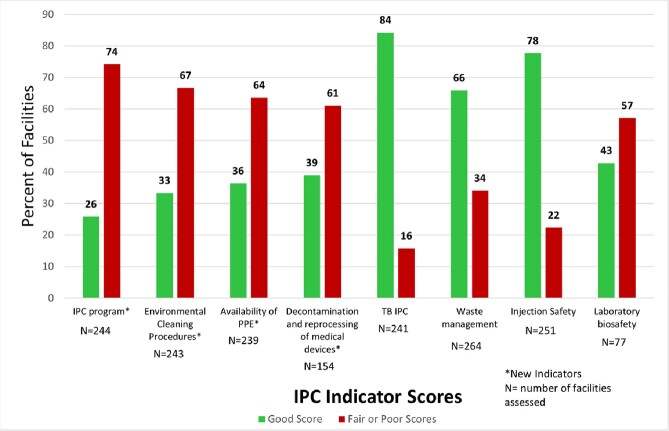
Infection Prevention and Control (IPC) Indicator Scores from PEPFAR Panorama for Quarter 1 (October 1 – December 31, 2022)

**Conclusion:**

Unknown IPC weaknesses were identified in PEPFAR supported facilities. PEPFAR requires that facilities with fair or poor compliance be reassessed within six months to monitor improvements. Strengthening IPC leadership is a priority. Improvement activities will require collaboration between facilities, implementing partners, and Ministries of Health (MOH). Future plans include increasing compliance with conducting required IPC indicator assessments; timely, data driven identification of gaps and advancements towards IPC standards; and developing guidance and resources to facilitate improvements. MOHs can leverage data to inform priorities and design context-specific interventions.

**Disclosures:**

**All Authors**: No reported disclosures

